# 1,2-Bis[bis­(methyl­sulfan­yl)methyl­ene]hydrazine

**DOI:** 10.1107/S1600536808014608

**Published:** 2008-05-21

**Authors:** Mohamed Driss, Meriem Toumi, Fatma Ben Amor, Ahmed Driss, Khaled Boujlel

**Affiliations:** a Laboratoire de Matériaux et Cristallochimie, Faculté des Sciences de Tunis, Université de Tunis El Manar, 2092 El Manar I Tunis, Tunisia; b Laboratoire de Chimie Analytique et Electrochimie, Faculté des Sciences de Tunis, Université de Tunis El Manar, 2092 El Manar I Tunis, Tunisia

## Abstract

The title compound, C_6_H_12_N_2_S_4_, was obtained as a by-product (8%) during the reaction of the electrogenerated cyano­methyl anion with phenyl­amine, carbon disulfide and methyl iodide. The mol­ecule, with the exception of 8 H atoms, lies on a crystallographic mirror plane and is arranged around an inversion centre located at the mid-point of the N—N bond.

## Related literature

For related literature, see: Pomes Hernandez *et al.* (1996 ▶); Toumi *et al.* (2007 ▶).
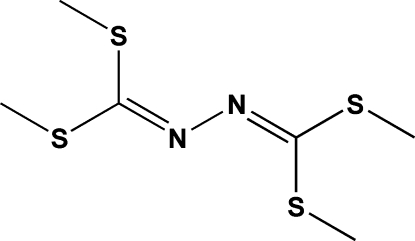


## Experimental

### 

#### Crystal data

C_6_H_12_N_2_S_4_
*M* *_r_* = 240.42
Monoclinic, 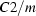

*a* = 10.683 (2) Å
*b* = 7.193 (1) Å
*c* = 8.309 (2) Å
β = 117.66 (2)°
*V* = 565.5 (2) Å^3^
*Z* = 2
Mo *K*α radiation
μ = 0.79 mm^−1^
*T* = 298 (2) K
0.50 × 0.29 × 0.22 mm

#### Data collection

Enraf–Nonius CAD-4 EXPRESS diffractometer
Absorption correction: ψ scan (North *et al.*, 1968 ▶) *T*_min_ = 0.79, *T*_max_ = 0.84
1994 measured reflections
885 independent reflections
701 reflections with *I* > 2σ(*I*)
*R*_int_ = 0.027
2 standard reflections frequency: 120 min intensity decay: 2%

#### Refinement

*R*[*F* ^2^ > 2σ(*F* ^2^)] = 0.032
*wR*(*F* ^2^) = 0.088
*S* = 1.06
885 reflections
37 parameters
H-atom parameters constrained
Δρ_max_ = 0.26 e Å^−3^
Δρ_min_ = −0.22 e Å^−3^

### 

Data collection: *CAD-4 EXPRESS* (Duisenberg, 1992 ▶; Macíček & Yordanov, 1992 ▶); cell refinement: *CAD-4 EXPRESS*; data reduction: *XCAD4* (Harms & Wocadlo, 1995 ▶); program(s) used to solve structure: *SHELXS97* (Sheldrick, 2008 ▶); program(s) used to refine structure: *SHELXL97* (Sheldrick, 2008 ▶); molecular graphics: *ORTEPIII* (Burnett & Johnson, 1996 ▶) and *ORTEP-3 for Windows* (Farrugia, 1997 ▶); software used to prepare material for publication: *WinGX* (Farrugia, 1999 ▶).

## Supplementary Material

Crystal structure: contains datablocks I, global. DOI: 10.1107/S1600536808014608/dn2348sup1.cif

Structure factors: contains datablocks I. DOI: 10.1107/S1600536808014608/dn2348Isup2.hkl

Additional supplementary materials:  crystallographic information; 3D view; checkCIF report

## Figures and Tables

**Fig. 1.**
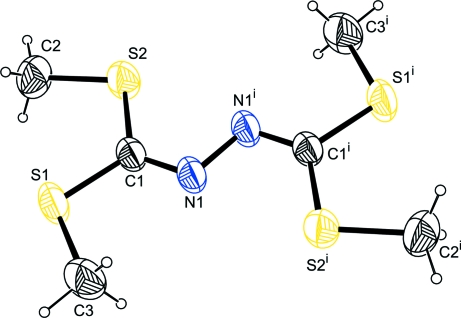
Molecular view of the title compound with the atom-labelling scheme. Thermal ellipsoids are drawn at the 50% probability level. H atoms are represented as small spheres of arbitrary radii. [Symmetry code: (i) 1-x, 1-y, 1-z]

## supplementary crystallographic information

### Comment

The structure is built up of C_6_H_12_N_2_S_4_ molecules which lie on mirror planes perpendicular to [0 1 0] direction. The molecule is centrosymetric around N1—N1^i^ bond (i: 1 - *x*, 1 - *y*, 1 - *z*) (figure 1).

The asymetric unit is built up by a nitrogen atom N1 bonded to C1 carbon which is bonded to sulfur atoms S1 and S2, each of them is bonded to a carbon atom. The values of the bond distance C1=N1 (1.283 Å), the bond distance average C—S (1.782 (3) Å), the angle S1C1S2 (117.6 (1)°) and the angles average CSC (103 (1)°) agree with those found in compounds having such bonds (Pomes Hernandez *et al.*, 1996; Toumi *et al.*, 2007). The deviations of H1 and H3 atoms from the plane of the molecule are 0.79 (2)Å and 0.71 (2)Å respectively.

### Experimental

The title compound was obtained from the electrolysis of a mixture of acetonitrile (ACN) (70 ml) and hexamethylphosphorotriamide (HMPT) (6 ml), under galvanostatic conditions (I = 105 mA, Q = 1,2 F/mol), in the presence of tetraethylammonium hexafluorophosphate (TEAPF6) (350 mg) as supporting electrolyte. At the end of the electrolysis, the hydrazone (diarylhydrazone) was added and the solution was kept under continuous stirring for one hour, the carbon disulfide was added (20 mmol) after 15 minutes of stirring and finally the methyl iodide was introduced and the solution was kept under stirring over night. After the removal of acetonitrile under reduced pressure, the residue was quenched with water and extracted with diethyl ether. The resulting product was chromatographed on silica gel (mesh 60, ethyl acetate / cyclohexane 1 / 9) to afford a pure product (yield 8%). Crystals suitable for X-ray analysis were grown by slow evaporation of dichloromethane solution.

The title compound was characterized by ^1^H, ^13^C NMR and MS spectra analysis. ^1^H NMR (CDCl_3_, 300 MHz): 2.45 (s, 6 H, CH_3_) and 2.52 (s, 6 H, CH_3_). ^13^C NMR (CDCl_3_, 75 MHz): 13.8 (CH_3_); 15.3 (CH_3_) and 163.5 (C=N). MS (EI) (%): m/*z* = 240 (35 / *M*+.); 193 (20); (*m*-MeS); 120 (100); (*M*-(MeS)2 C=N).

### Refinement

All H atoms attached to C atoms were fixed geometrically and treated as riding with C—H = 0.96 Å (methyl) with U_iso_(H) = 1.5U_eq_(C).

### Crystal data

**Table:**

C_6_H_12_N_2_S_4_	*F*_000_ = 252
*M**_r_* = 240.42	*D*_x_ = 1.412 Mg m^−^^3^
Monoclinic, *C*2/*m*	Mo *K*α radiation λ = 0.71073 Å
Hall symbol: -C 2y	Cell parameters from 25 reflections
*a* = 10.683 (2) Å	θ = 10–15º
*b* = 7.193 (1) Å	µ = 0.79 mm^−^^1^
*c* = 8.309 (2) Å	*T* = 298 (2) K
β = 117.66 (2)º	Plate, yellow
*V* = 565.5 (2) Å^3^	0.50 × 0.29 × 0.22 mm
*Z* = 2	

### Data collection

**Table:**

Enraf–Nonius CAD-4 EXPRESS diffractometer	*R*_int_ = 0.027
Radiation source: fine-focus sealed tube	θ_max_ = 30.0º
Monochromator: graphite	θ_min_ = 2.8º
*T* = 298(2) K	*h* = −14→14
ω/2θ scans	*k* = −1→10
Absorption correction: ψ scan(North *et al.*, 1968)	*l* = −11→11
*T*_min_ = 0.79, *T*_max_ = 0.84	2 standard reflections
1994 measured reflections	every 120 min
885 independent reflections	intensity decay: 2%
701 reflections with *I* > 2σ(*I*)	

### Refinement

**Table:**

Refinement on *F*^2^	Secondary atom site location: difference Fourier map
Least-squares matrix: full	Hydrogen site location: inferred from neighbouring sites
*R*[*F*^2^ > 2σ(*F*^2^)] = 0.032	H-atom parameters constrained
*wR*(*F*^2^) = 0.088	*w* = 1/[σ^2^(*F*_o_^2^) + (0.0422*P*)^2^ + 0.172*P*] where *P* = (*F*_o_^2^ + 2*F*_c_^2^)/3
*S* = 1.06	(Δ/σ)_max_ = 0.001
885 reflections	Δρ_max_ = 0.26 e Å^−^^3^
37 parameters	Δρ_min_ = −0.22 e Å^−^^3^
Primary atom site location: structure-invariant direct methods	Extinction correction: none

### Special details

**Table:**

Geometry. All esds (except the esd in the dihedral angle between two l.s. planes) are estimated using the full covariance matrix. The cell esds are taken into account individually in the estimation of esds in distances, angles and torsion angles; correlations between esds in cell parameters are only used when they are defined by crystal symmetry. An approximate (isotropic) treatment of cell esds is used for estimating esds involving l.s. planes.
Refinement. Refinement of *F*^2^ against ALL reflections. The weighted *R*-factor *wR* and goodness of fit *S* are based on *F*^2^, conventional *R*-factors *R* are based on *F*, with *F* set to zero for negative *F*^2^. The threshold expression of *F*^2^ > σ(*F*^2^) is used only for calculating *R*-factors(gt) *etc*. and is not relevant to the choice of reflections for refinement. *R*-factors based on *F*^2^ are statistically about twice as large as those based on *F*, and *R*- factors based on ALL data will be even larger.

### Fractional atomic coordinates and isotropic or equivalent isotropic displacement parameters (Å^2^)

**Table:**

	*x*	*y*	*z*	*U*_iso_*/*U*_eq_	
S1	0.83746 (5)	0.5000	0.78339 (7)	0.0648 (2)	
S2	0.58364 (5)	0.5000	0.85038 (7)	0.0605 (2)	
N1	0.57245 (14)	0.5000	0.5218 (2)	0.0493 (4)	
C1	0.65298 (17)	0.5000	0.6951 (2)	0.0435 (4)	
C2	0.7394 (3)	0.5000	1.0710 (3)	0.0695 (7)	
H2A	0.7109	0.5000	1.1650	0.104*	
H2B	0.7948	0.6090	1.0824	0.104*	
C3	0.8586 (3)	0.5000	0.5812 (4)	0.0732 (8)	
H3A	0.9574	0.5000	0.6140	0.110*	
H3B	0.8148	0.6090	0.5107	0.110*	

### Atomic displacement parameters (Å^2^)

**Table:**

	*U*^11^	*U*^22^	*U*^33^	*U*^12^	*U*^13^	*U*^23^
S1	0.0283 (2)	0.1133 (6)	0.0464 (3)	0.000	0.01185 (19)	0.000
S2	0.0414 (3)	0.0984 (5)	0.0439 (3)	0.000	0.0217 (2)	0.000
N1	0.0286 (6)	0.0772 (12)	0.0392 (7)	0.000	0.0132 (6)	0.000
C1	0.0302 (7)	0.0566 (11)	0.0413 (8)	0.000	0.0146 (6)	0.000
C2	0.0619 (13)	0.1011 (19)	0.0380 (9)	0.000	0.0168 (9)	0.000
C3	0.0475 (11)	0.116 (2)	0.0656 (13)	0.000	0.0339 (10)	0.000

### Geometric parameters (Å, °)

**Table:**

S1—C1	1.7550 (18)	N1—N1^i^	1.417 (3)
S1—C3	1.796 (3)	C2—H2A	0.9600
S2—C1	1.7609 (19)	C2—H2B	0.9600
S2—C2	1.816 (2)	C3—H3A	0.9600
N1—C1	1.290 (2)	C3—H3B	0.9600
			
C1—S1—C3	102.32 (10)	S2—C2—H2A	109.5
C1—S2—C2	103.88 (10)	S2—C2—H2B	109.5
C1—N1—N1^i^	111.67 (18)	H2A—C2—H2B	109.5
N1—C1—S1	120.29 (14)	S1—C3—H3A	109.5
N1—C1—S2	121.90 (13)	S1—C3—H3B	109.5
S1—C1—S2	117.81 (10)	H3A—C3—H3B	109.5

Symmetry codes: (i) −*x*+1, −*y*+1, −*z*+1.
